# Optical biomarkers for Alzheimer's disease prediction in a heterogenous clinical cohort

**DOI:** 10.1002/alz70856_100770

**Published:** 2025-12-25

**Authors:** George Devitt, Sofia Michopoulou, Angus Prosser, Boyd CP Ghosh, Amritpal Mudher, Christopher Kipps, Sumeet Mahajan

**Affiliations:** ^1^ University Hospital Southampton NHS Foundation Trust, Southampton, United Kingdom; ^2^ University of Southampton, Southampton, United Kingdom; ^3^ Wessex Neurological Centre, University Hospital Southampton, Southampton, United Kingdom

## Abstract

**Background:**

Reported accuracies of clinical Alzheimer's Disease (AD) diagnosis vary widely due to diagnostic inconsistency and patient heterogeneity. Raman spectroscopy is a label‐free, laser‐based method that can rapidly provide chemically rich information from biofluids. Despite this, AD classification using Raman spectroscopy has not yet been tested in a cohort representative of the clinical setting with appropriate statistical power.

**Method:**

Cerebrospinal fluid (CSF) samples were cross‐sectionally assessed from a mixed clinical cohort of patients (*N* = 141) using Raman Spectroscopy. Raman spectra from AD (*n* = 66) and non‐AD (*n* = 75) patients were divided into training at testing sets at a ratio of 80:20 and machine‐learning (ML) models were trained, optimized and evaluated for AD classification. ML models included a support‐vector machine (SVM) and a convolutional neural network (CNN). Area under the receiver operating characteristic curve (AUROC) analysis was used to assess classifier performance. To explain AD classification, key spectral features were extracted using the Mann–Whitney U test per Raman shift and spectral regions were integrated to provide univariate features that were corelated to ATN biomarker status.

**Result:**

Optimized ML models generalized well in the AD cohort with 93% classification accuracy observed for the SVM model (AUROC = 0.92, sensitivity = 0.92, specificity = 0.93). There was a strong correlation between the classifier score and patient ATN biomarker status. Patients with other neurodegenerative diseases were correctly classified as non‐AD group including corticobasal degeneration (CBD), dementia with Lewy bodies (DLB), frontotemporal dementia (FTD), and Parkinson's disease. Important features for AD classification primarily included protein‐derived aromatic amino acids particularly phenylalanine and tyrosine. 9 features were extracted and moderate correlations were observed for each feature with ATN biomarker status demonstrating the importance of the composite spectrum for classification.

**Conclusion:**

Our results demonstrate that Raman spectroscopy can accurately identify AD using CSF from a mixed clinical cohort in which other causes of dementia are prevalent. Although larger cross‐sectional studies are required to assess whether accuracy is retained at a population level, establishing the utility of RS in a clinical population is an important first step towards the translation of optical biomarkers for supporting AD diagnosis in the future.

